# Supplementary material for: Insulin Resistance Is Associated with Reduced Food Odor Sensitivity across a Wide Range of Body Weights
Source: Nutrients. 2020 Jul 24;12(8):2201. doi: 10.3390/nu12082201 (PMC7468861; doi:10.3390/nu12082201)
Supplement: Supplementary file 1 [file nutrients-12-02201-s001.pdf]

Supplementary

Figure S1

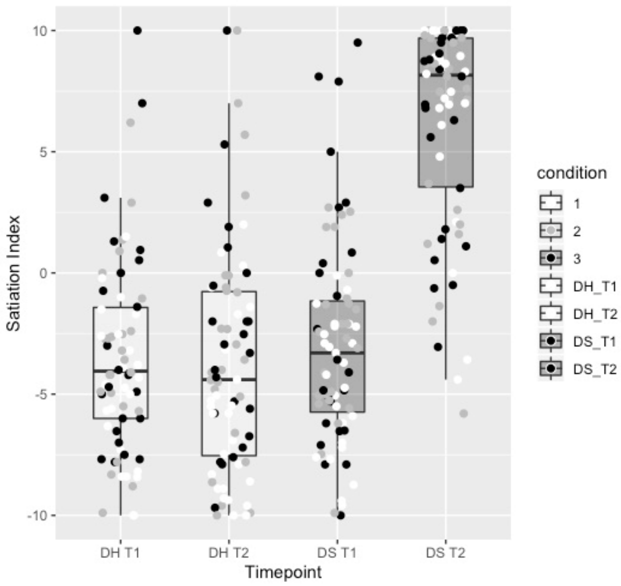

Figure S1 Sanity Check: Subjective hunger depicted by visual analogue ratings. A satiation index was computed by subtracting hunger ratings from fullness ratings at each time point of internal state ratings on visual analog scales (Sun et al., 2016). A high positive index can be interpreted as very sated and a high negative index as very hungry. Because of heterogeneous variances, a Wilcoxon test was performed with satiety index pre- and postprandial under two conditions (day hunger vs. day sated). The Wilcoxon Signed-Ranks Test indicated for the sated condition significantly higher median post-test than the median pre-test scores ($Z=-7.292$, $p<.001$), whereas it was the other way around for the hungry condition ($Z=-2.304$, $p=.021$). Thus, as expected, participants felt sated after meal in the sated condition and perceived more hunger or less fullness in the hungry condition after the break without a meal.

Figure S2

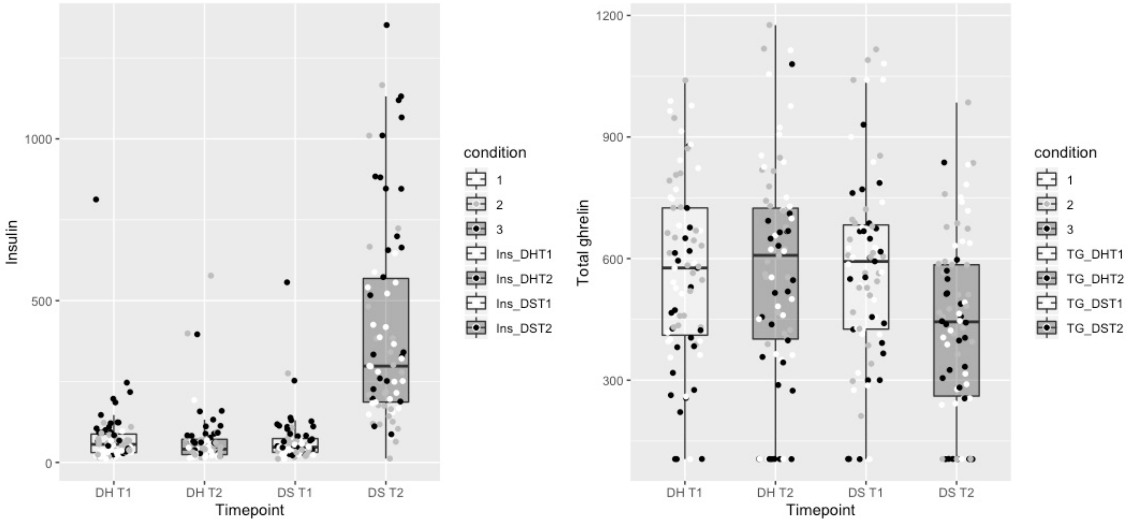

Figure S2 Sanity Check: Physiological hunger depicted by pre- and postprandial insulin and ghrelin levels. Wilcoxon Signed Ranks test over all groups for insulin and total ghrelin levels before and after meal at both test days as proxies of depicting physiological hunger. As expected we could show a significant increase of insulin levels in the sated condition after meal intake ($Z=-6.931$, $p<.001$) and no change of insulin levels in the hungry condition ($Z=-1.447$, $p=.148$). As expected total ghrelin levels decreased in the sated condition after meal intake ($Z=-4.851$, $p<.001$) and did not change in the hungry condition ($Z=-1.686$, $p=.092$).

Table S1 Olfactory sensitivity and sex/cycle phase in women

	Male	Female	Menstrual cycle phase (in women)	
			Luteal phase (n=15)	Follicular phase (n=22)
Average ODT	8.31 ± 1.13<br>5.33 – 10.17	8.93 ± 1.27<br>4.89 – 10.89	8.77 ± 1.52<br>4.89 – 10.89	9.04 ± 1.09<br>6.50 – 10.83

Table S2 Hormonal parameters in weight groups and correlation with BMI

	Group level					Correlation analysis	
	NW	OW	OB	Test	sig	Corr. with BMI	sig
Insulin	37.01 ± 16.96 (10.60 – 86.95)	54.85 ± 24.74 (18.85 – 108.80)	89.34 ± 42.60 (25.10 – 179.90)	ANOVA F = 19.122/df=2	*p* < .001	.619**	*p* < .001
Post hoc: (Tukey) NW+OB (p<.001); OW+OB (p=.001)							
IR	.74 ± .30 (.44 – 1.66)	1.06 ± .45 (.45 – 2.04)	1.67 ± .78 (.48 – 3.32)	K-W H test F = 24.228/df=2	*p* < .001	.601**	*p* < .001
Leptin	6.19 ±3.92 (1.20 – 15.00)	10.88 ± 10.04 (.80 – 33.20)	18.61 ± 15.02 (3.80 – 52.20)	ANOVA F = 24.76/df=2	*p* < .001	.476**	*p* < .001
Post hoc: (Games Howell, covariate: sex) NW+OB (p=.005)							
Total ghrelin	604.35 ± 238.32 (182.75 – 1035.00)	626.46 ± 186.43 (366.50 – 1078.00)	478.75 ± 204.12 (105.50 – 903.50)	ANOVA F = 3.474/df=2	*p* = .036	-.226*	*p* = .026
Post hoc: (Tukey) OW + OB (p=.030)							
AG	11.62 ± 7.06 (1.71 – 25.68)	13.85 ± 9.98 (2.27 – 45.14)	15.05 ± 16.63 (1.00 – 59.66)	ANOVA F = .526/df=2	*p* = .593	.037	*p* = .760
UAG	21.40 ± 16.25 (2.71 – 58.48)	16.22 ± 8.67 (3.27 – 38.27)	19.75 ± 11.21 (3.00 – 41.20)	ANOVA F = 1.126/df=2	*p* = .330	-.004	*p* = .973
AG/UAG ratio	1.24 ± 1.51 (.03 – 5.92)	1.48 ± 1.52 (.10 – 5.78)	1.25 ± 1.40 (.03 – 4.90)	ANOVA	*p* = .818	.012	*p* = .923

F =
.202/df=2

Table S3 Mediation analysis. Relationship between BMI and olfaction via hormonal parameters

	Estimate	SE/MSE	t/F	p	R²	Lower 95% CI	Upper 95% CI
FOOD ODT general via metabolic state (BMI as x variable)							
Path a**							
M1: IR	**.61**	.68	12.53	**<.001***	.37		
M2: Leptin	**.83**	.30	48.98	*****	.70		
M3: AG/UAG ratio	.28	1.00	1.75	**<.001***	.08		
M4: TG	**.31**	.87	2.24	*****	.09		
				.1656			
				.0925			
Path b**					.		
Model Summary	.40	.94	1.64	.1414	.16		
X: BMI	.19	.20	.93	.3560		-.216	.591
M1: IR	**-.42**	.15	-2.77	**.0075***		**-.717**	**-.115**
M2: Leptin	-.06	.22	-.29	*****		-.509	.380
M3: AG/UAG ratio	-.10	.12	-.80	.7716		-.343	.148
M4: TG	-.20	.13	-1.47	.4312		-.461	.070
Co1: Sex	.50	.36	1.37	.1458		-.229	1.221
				.1762			
Total effect	-.09	.13	-.70	.4870		-.348	.167
Direct effect	.19	.20	.93	.3560		-.216	.591
Indirect effect**							
IR	**-.26**	.10				**-.496**	**-.087**
Leptin	-.04	.15				-.385	.220
AG/UAG ratio	-.02	.03				-.090	.050
TG	.04	.04				-.016	.130
FOOD ODT Change via metabolic state (BMI as x variable)							
Path a**							
M1: IR	**.61**	.68	12.53	**<.001***	.37		
M2: Leptin	**.83**	.30	48.98	*****	.70		
M3: AG/UAG ratio	.28	1.00	1.75	**<.001***	.08		
M4: TG	**.31**	.87	2.24	*****	.09		
				.1656			
				.0925			
Path b**							
Model Summary	**.52**	2.32	2.32	**.0056***	.27		
X: BMI	**.71**	.32	2.23	*****		**.074**	**1.340**
M1: IR	**-.72**	.24	-3.06	**.0293***		**-1.194**	**-.250**
M2: Leptin	**-.72**	.35	-2.06	**.0033***		-1.414	-.019
M3: AG/UAG ratio	-.27	.19	-1.37	*****		-.649	.109
M4: TG	-.53	.21	-2.53	**.0443***		-.942	-.109
Co1: Sex	**1.49**	.57	2.61	.1745		**.348**	**2.621**
Co2: Meal ingested	-.39	.21	-1.89	**.0142***		-.803	.0234
				**.0114***			
				.0640			
Total effect	-.13	.22	-.61	.5471		-.563	.301

<u>Direct effect*</u>	**.71**	.32	2.23	**.0293***		**.074**	**1.340**
<u>Indirect effect**</u>							
IR	**-.44**	.16				**-.808**	**-.190**
Leptin	**-.44**	.22				**-.990**	**.117**
AG/UAG ratio	-.05	.06				-.183	.046
TG	-.10	.08				-.019	.292

Non FOOD ODT general via metabolic state (BMI as x variable)

<u>Path a**</u>							
M1: IR	**.61**	.68	12.53	**<.001***	.37		
M2: Leptin	**.83**	.30	48.98	*****	.70		
M3: AG/UAG ratio	.28	1.01	1.75	**<.001***	.08		
M4: TG	**.31**	.87	2.24	*****	.09		
				.1656			
				**.0435***			
<u>Path b</u>							
Model Summary	.32	.99	.95	.4777	.10		
X: BMI	.16	.21	.77	.4431		-.254	.574
M1: IR	-.22	.15	-1.41	.1635		-.526	.091
M2: Leptin	-.02	.23	-.09	.9320		-.475	.436
M3: AG/UAG ratio	-.11	.13	-.89	.3768		-.364	.140
M4: TG	.07	.14	.55	.5839		-.197	.347
Co1: Sex	.45	.37	1.20	.2356		-.298	.188
<u>Total effect</u>	-.02	.13	-.16	.8751		-.273	.233
<u>Direct effect</u>	.16	.21	.77	.4431		-.254	.574
<u>Indirect effect</u>							
IR	-.13	.09				-.331	.030
Leptin	-.01	.15				-.287	.309
AG/UAG ratio	-.02	.03				-.099	.043
TG	-.01	.03				-.080	.037

Non FOOD ODT Change via metabolic state (BMI as x variable)

<u>Path a**</u>							
M1: IR	**.61**	.68	12.53	**<.001***	.37		.
M2: Leptin	**.83**	.30	48.98	*****	.70		
M3: AG/UAG ratio	.28	1.01	1.75	**<.001***	.08		
M4: TG	**.31**	.87	2.24	*****	.09		
				.1656			
				**.0435***			
<u>Path b</u>							
Model Summary	.29	3.23	.80	.5915	.09		
X: BMI	.47	.37	1.25	.2177		-.282	1.213
M1: IR	-.30	.28	-1.08	.2836		-.859	.256
M2: Leptin	-.64	.41	-1.56	.1248		-1.463	.183
M3: AG/UAG ratio	.13	.23	.56	.5766		-.327	.582
M4: TG	.10	.25	.42	.6736		-.387	.695
Co1: Sex	.86	.67	1.29	.2029		-.478	2.201
Co2: Meal ingested	-.15	.24	-.63	.5332		-.640	.335
<u>Total effect</u>	-.11	.23	-.476	.6359		-.571	.352
<u>Direct effect</u>	.47	.37	1.25	.2177		-.282	1.213
<u>Indirect effect</u>							
IR	-.19	.17				-.541	.123

Leptin	-.39	.32				-1.061	.214
AG/UAG ratio	.02	.08				-.135	.208
TG	-.02	.06				-.156	.088
FOOD ODT general via metabolic state (WHR as x variable)							
Path a**							
M1: IR	**.61**	.68	12.53	**<.001***	.37		
M2: Leptin	**.83**	.30	48.98	*	.70		
M3: AG/UAG ratio	.28	1.00	1.75	**<.001***	.08		
M4: TG	**.31**	.87	2.24	*	.09		
				.1656			
				.0925			
Path b**					.		
Model Summary	.40	.94	1.64	.1414	.16		
X: BMI	.19	.20	.93	.3560		-.216	.591
M1: IR	**-.42**	.15	-2.77	**.0075***		**-.717**	**-.115**
M2: Leptin	-.06	.22	-.29	*		-.509	.380
M3: AG/UAG ratio	-.10	.12	-.80	.7716		-.343	.148
M4: TG	-.20	.13	-1.47	.4312		-.461	.070
Co1: Sex	.50	.36	1.37	.1458		-.229	1.221
				.1762			
Total effect	-.09	.13	-.70	.4870		-.348	.167
Direct effect	.19	.20	.93	.3560		-.216	.591
Indirect effect**							
IR	**-.26**	.10				**-.496**	**-.087**
Leptin	-.04	.15				-.385	.220
AG/UAG ratio	-.02	.03				-.090	.050
TG	.04	.04				-.016	.130
FOOD ODT Change via metabolic state (WHR as x variable)							
Path a**							
M1: IR	**.61**	.68	12.53	**<.001***	.37		
M2: Leptin	**.83**	.30	48.98	*	.70		
M3: AG/UAG ratio	.28	1.00	1.75	**<.001***	.08		
M4: TG	**.31**	.87	2.24	*	.09		
				.1656			
				.0925			
Path b**							
Model Summary	**.52**	2.32	2.32	**.0056***	.27		
X: BMI	**.71**	.32	2.23	*		**.074**	**1.340**
M1: IR	**-.72**	.24	-3.06	**.0293***		**-1.194**	**-.250**
M2: Leptin	**-.72**	.35	-2.06	**.0033***		-1.414	-.019
M3: AG/UAG ratio	-.27	.19	-1.37	*		-.649	.109
M4: TG	-.53	.21	-2.53	**.0443***		-.942	-.109
Co1: Sex	**1.49**	.57	2.61	.1745		**.348**	**2.621**
Co2: Meal ingested	-.39	.21	-1.89	**.0142***		-.803	.0234
				**.0114***			
				.0640			
Total effect	-.13	.22	-.61	.5471		-.563	.301
Direct effect*	**.71**	.32	2.23	**.0293***		**.074**	**1.340**

Indirect effect**							
IR	**-.44**	.16				**-.808**	**-.190**
Leptin	**-.44**	.22				**-.990**	**.117**
AG/UAG ratio	-.05	.06				-.183	.046
TG	-.10	.08				-.019	.292

Non FOOD ODT general via metabolic state (WHR as x variable)							
Path a**							
M1: IR	**.61**	.68	12.53	**<.001***	.37		
M2: Leptin	**.83**	.30	48.98	*****	.70		
M3: AG/UAG ratio	.28	1.01	1.75	**<.001***	.08		
M4: TG	**.31**	.87	2.24	*****	.09		
				.1656			
				**.0435***			
Path b							
Model Summary	.32	.99	.95	.4777	.10		
X: BMI	.16	.21	.77	.4431		-.254	.574
M1: IR	-.22	.15	-1.41	.1635		-.526	.091
M2: Leptin	-.02	.23	-.09	.9320		-.475	.436
M3: AG/UAG ratio	-.11	.13	-.89	.3768		-.364	.140
M4: TG	.07	.14	.55	.5839		-.197	.347
Co1: Sex	.45	.37	1.20	.2356		-.298	.188
Total effect	-.02	.13	-.16	.8751		-.273	.233
Direct effect	.16	.21	.77	.4431		-.254	.574
Indirect effect							
IR	-.13	.09				-.331	.030
Leptin	-.01	.15				-.287	.309
AG/UAG ratio	-.02	.03				-.099	.043
TG	-.01	.03				-.080	.037
Non FOOD ODT Change via metabolic state (WHR as x variable)							
Path a**							
M1: IR	**.61**	.68	12.53	**<.001***	.37		.
M2: Leptin	**.83**	.30	48.98	*****	.70		
M3: AG/UAG ratio	.28	1.01	1.75	**<.001***	.08		
M4: TG	**.31**	.87	2.24	*****	.09		
				.1656			
				**.0435***			
Path b							
Model Summary	.29	3.23	.80	.5915	.09		
X: BMI	.47	.37	1.25	.2177		-.282	1.213
M1: IR	-.30	.28	-1.08	.2836		-.859	.256
M2: Leptin	-.64	.41	-1.56	.1248		-1.463	.183
M3: AG/UAG ratio	.13	.23	.56	.5766		-.327	.582
M4: TG	.10	.25	.42	.6736		-.387	.695
Co1: Sex	.86	.67	1.29	.2029		-.478	2.201
Co2: Meal ingested	-.15	.24	-.63	.5332		-.640	.335

Total effect	-.11	.23	-.476	.6359	-.571	.352
Direct effect	.47	.37	1.25	.2177	-.282	1.213
Indirect effect						
IR	-.19	.17			-.541	.123
Leptin	-.39	.32			-1.061	.214
AG/UAG ratio	.02	.08			-.135	.208
TG	-.02	.06			-.156	.088

Table S4: Overview: Olfactory sensitivity between participants with normal weight and obesity for age and BMI.

Study	Normal weight group			Obese group			Finding	Comment
	N	Age	BMI	n	Age	BMI		
**Fernandez-Garcia (2017)**	77	27.1 ±7.3	21.6 ±1.7	28	46.4 ±12.2	35.2 ±2.6	Lower ODT (n-butanol)	
**Fernandez-Aranda (2016)**	36	37.3 ±5.9	22.4 ±2.6	59	37.5 ±8.7	42.7 ±6.6	Lower ODT (n-butanol)	
**Pastor (2016)**	70	27.4 ±7.36	21.7 ±1.62	26	47.3 ±11.1	35.5 ±2.53	Lower ODT (n-butanol)	
**Stafford (2015)**	20	19.4 ±.3	20.3	20	20.4 ±.4	31.3 ±.3	Higher ODT (chocolate)	
**Skrandies (2015)**				7	35.5	34.4	Lower ODT in higher BMI participants	Compared to underweight & normal weight
